# Soluble CD137 Ameliorates Acute Type 1 Diabetes by Inducing T Cell Anergy

**DOI:** 10.3389/fimmu.2019.02566

**Published:** 2019-11-07

**Authors:** Arata Itoh, Lorenzo Ortiz, Kritika Kachapati, Yuehong Wu, David Adams, Kyle Bednar, Shibabrata Mukherjee, Claire Chougnet, Robert S. Mittler, Yi-Guang Chen, Laurence Dolan, William M. Ridgway

**Affiliations:** ^1^Division of Immunology, Allergy and Rheumatology, University of Cincinnati College of Medicine, Cincinnati, OH, United States; ^2^Division of Immunobiology, Cincinnati Children's Hospital Medical Center, Cincinnati, OH, United States; ^3^Department of Surgery, Emory University, Atlanta, GA, United States; ^4^Emory Vaccine Center, Atlanta, GA, United States; ^5^Department of Pediatrics, Medical College of Wisconsin, Milwaukee, WI, United States; ^6^Division of Endocrinology, Cincinnati Children's Hospital Medical Center, Cincinnati, OH, United States

**Keywords:** type 1 diabetes, autoimmunity, T cells, T cell anergy, soluble CD137

## Abstract

We show here that soluble CD137 (sCD137), the alternately spliced gene product of *Tnfsfr9*, effectively treats acute type 1 diabetes (T1D) in nonobese diabetic (NOD) mice. sCD137 significantly delayed development of end-stage disease, preserved insulin+ islet beta cells, and prevented progression to end-stage T1D in some mice. We demonstrate that sCD137 induces CD4+ T cell anergy, suppressing antigen-specific T cell proliferation and IL-2/IFN-γ secretion. Exogenous IL-2 reversed the sCD137 anergy effect. sCD137 greatly reduces inflammatory cytokine production by CD8 effector memory T cells, critical mediators of beta cell damage. We demonstrate that human T1D patients have decreased serum sCD137 compared to age-matched controls (as do NOD mice compared to NOD congenic mice expressing a protective *Tnfsfr9* allele), that human sCD137 is secreted by regulatory T cells (Tregs; as in mice), and that human sCD137 induces T cell suppression in human T cells. These findings provide a rationale for further investigation of sCD137 as a treatment for T1D and other T cell–mediated autoimmune diseases.

## Introduction

Type 1 diabetes (T1D) is a complex autoimmune disease resulting from specific immunological destruction of pancreatic insulin-producing beta cells, leading to hyperglycemia, and ketoacidosis (1, 2). Nonobese diabetic (NOD) mice are a well-established animal model of T1D that reflects many of the same features as human T1D (3, 4). NOD mice spontaneously develop T1D (typically ~80% of females spontaneously develop T1D by 30 weeks in many colonies), mediated by autoreactive CD4 and CD8 T cells recognizing many of the same autoantigens found in the human disease. NOD mice also demonstrate genetic control of T1D with key contributions from genes that are implicated in human disease, including MHC class II genes (3). CD4 and CD8 T cells, in combination with environmental factors and genetic background, play an essential role in beta cell destruction in both NOD mice and humans (5, 6). In NOD mice, dysregulation of both effector and regulatory T cells (Tregs) is well-described (7).

Wicker et al. originally established that the B10 chromosome 4 genetic region *Idd9.3*, which includes the candidate gene *Tnfsfr9* (expressing CD137, also known as 4-1bb), protects from T1D in NOD *Idd9.3* congenic mice (8). We published that treatment with an agonistic CD137 antibody prevented T1D in NOD mice, at least partly by targeting and increasing the numbers of the CD4+CD25+CD137+ Treg subset (9). We then showed that the protective B10 *Idd9.3* allele was associated with increased numbers of CD4+CD25+CD137+ Tregs, which were functionally superior to CD25+ Tregs (10). We further showed that CD137+ Tregs produce an alternately spliced, soluble form of CD137, “sCD137,” and that NOD mice had a decreased serum level of sCD137 compared to protected NOD *Idd9.3* congenic mice (10, 11). We produced recombinant mouse sCD137, demonstrated that it formed a homo-dimer, and showed that sCD137 directly suppresses effector CD4+CD25– and CD8 T cell proliferation in an APC-independent but CD137 ligand (CD137L)–dependent manner (11, 12). Finally, “restoring” serum levels by administration of recombinant sCD137 into NOD mice significantly prevented autoimmune diabetes compared with control treatment (11).

Although these results showed a suppressive effect of sCD137 on T cells, the mechanism of this effect was unclear. In addition, “prevention” of T1D (especially in NOD mice) is much different (and much easier to accomplish) than therapeutic efficacy in acute disease. Finally, we had not yet demonstrated any relevance of this work to human T1D. We address these issues in the current manuscript and show that (1) recombinant sCD137 acts by inducing antigen-specific T cell anergy; (2) sCD137 can ameliorate acute T1D; and (3) human T1D patients show a deficit of serum sCD137 similar to that seen in NOD mice, human Tregs are the main immune cell source of sCD137 just as in mice, and human sCD137 suppresses human T cell proliferation. These results support further exploration of sCD137 as a novel treatment approach in human T1D and other T cell–mediated autoimmune diseases.

## Materials and Methods

### Mice

NOD and NOD BDC2.5 transgenic mice were bred and maintained under specific pathogen-free conditions, and all procedures involving mice were conducted in accordance with the institutional animal care guidelines at the University of Cincinnati College of Medicine Laboratory Animal Medical Services.

### Purification of sCD137

Recombinant *Mus musculus* sCD137 was purified as previously described (9). Briefly, HEK293 cells were stably transduced with a lentiviral vector, LeGO-iG2-sCD137, expressing recombinant mouse sCD137 cDNA from the construct's SFFV promoter. Secreted sCD137 protein was purified from the culture supernatants using anti-CD137 antibody (clone: 3H3) affinity chromatography. After elution from the column, purified protein was dialyzed against 2 × 4 L of 1×TBS, then 2 × 4 L of 1×PBS, and then concentrated using Amicon Ultra-15 Ultra-cel 10 K centrifugal filters. The amount of purified sCD137 was determined by spectrophotometry and its specificity tested by binding to *M. musculus* CD137L-Myc-DDK protein expressed on the surface of HEK293 cells. SDS-PAGE and western blotting were used to confirm the dimeric state of active protein, as previously described (9). Prior to injection into mice, concentrated protein was thawed and diluted in sterile vehicle (1×PBS).

### *In vivo* Treatment of Diabetic NOD Mice With sCD137

Prediabetic female NOD mice were randomly assigned to either control or sCD137 treatment groups. These mice were assessed for diabetes onset using urine glucose paper testing (Tes-Tape, Nasco) and their glucose levels quantified with a standard one-step blood glucose meter. After onset of polyuria, and when two consecutive blood glucose measurements were between 200 and 250 mg/dl (group 1) or 250 and 300 mg/dl (group 2), mice were treated with sCD137 (120 μg/mice) intraperitoneally injected at day 0. At day 4 and after day 7, if their repeat BG was still 200 or 250 mg/dl, treatment with the same dose of sCD137 was continued weekly until they reached “end-stage diabetes,” defined as BG>500 mg/dl, or reached the study end point (10 weeks). Mice were excluded from the study (1) if they never developed T1D or (2) if their initial BG was not in the pre-specified range. The age of onset of diabetes in the female NOD mice in group 1 (initial BG 200–250) in the sCD137 and control groups was 24 ± 1 and 22 ± 3 weeks old of age, respectively (*p* = 0.4763). In group 2 (initial BG 250–300 mg/dl), the age of onset of the treatment and control groups was 24 ± 3 and 27 ± 2 weeks of age, respectively (*p* = 0.2797). For controls, group 1 used untreated NOD mice; this group of mice was also used as the control for another concurrent study, which has already been published (13). For group 2, control mice were treated with PBS i.p. at the same time points described above. At the end points, the pancreas was harvested and fixed in formalin for processing by the CCHMC Department of Anatomic Pathology. Pancreatic insulitis on H&E-stained slides was blindly scored as previously described (11, 13) (0: no visible infiltration; 1: peri-insulitis with a cell depth <5; 2: peri-insulitis with a cell depth >5; 3: insulitis with <50% islet infiltration; 4: insulitis with more than 50% islet infiltration; 5: islet scar). For quantification of islet immunofluorescence, insulin was stained with Insulin guinea pig primary antibody (Roche, cat. # 760-2655), and glucagon was stained with Glucagon rabbit primary antibody (Roche, cat. # 760-2644) overnight at 4°C, followed by staining using Texas-Red conjugated anti–guinea pig IgG and FITC-conjugated anti-rabbit IgG antibodies as secondary antibodies. Then, the nucleus was stained with DAPI (Vecta-shield with DAPI, Vector, cat. # H-1500) at the CCHMC Pathology Core. We imaged sCD137-treated mice that had not progressed to end-stage T1D and five randomly chosen control mice that reached end-stage diabetes. One pancreatic section per animal was stained with immunofluorescence. Whole slide digital images were created using a Leica Aperio AT2 digital slide scanner. Digital dual-immunofluorescence histology slides were analyzed using Photomerge (Adobe Photoshop) and Aperio ImageScope software (Leica Biosystems), used to count insulin and glucagon positive-staining regions per section.

### Antigen-Specific T Cell Proliferation Assay

CD4+CD25– effector T cells were magnetically isolated from 6- to 10-week-old female BDC2.5 transgenic female mice using the CD4+ T cell isolation kit followed by removal of CD25+ cells with the CD25+ microbeads kit (Myltenyi Biotec). Fifty thousand cells were co-cultured with antigen-presenting cells plus 10 nM of BDC2.5:insulin hybrid peptide [sequence: DLQTLALWSRMD (14)], in the presence or absence of 60 μg/ml of sCD137 for 4 days on a 96-well flat-bottom plate in triplicated manner. Cells were resuspended in 0.2 mL of T cell culture media (TCM), composed of RPMI-1640 media (Lonza) supplemented with 10% heat-inactivated FBS (GE Lifescience), 10 mM of HEPES (Gibco), 2 mM of L-glutamine (Gibco), 100 U/ml of penicillin/streptomycin (Gibco), 100 nM of sodium pyruvate (Gibco), non-essential amino acids (Gibco), and 150 μM of β-mercaptoethanol (Sigma). In some wells, 25 U/ml of recombinant mouse IL-2 (Invitrogen) was added at the start or 24 h after starting cell culture. In the last 18 h of cell culture, 1 μCi of [3H] thymidine was added to each well. Cells were harvested and counted using a β-scintillation counter.

### Flow Cytometry for Cell-Surface and Intracellular Phosphorylated Proteins

CD4+CD25– or CD8 T cells (0.5 × 10^5^) were negatively isolated with either a CD4 T cell isolation kit and CD25 microbeads (Miltenyi Biotec) or a CD8 T cell isolation kit (Miltenyi Biotec), respectively. Cells were resuspended in 0.2 mL of TCM and stimulated with CD3/28 dynabeads (cell-to-bead ratio 1:1) in the presence of sCD137 or rapamycin (10 nM at final concentration) for 24 h on a 96-well round-bottom plate. Harvested cells were stained with eFluor 450–conjugated rat anti-mouse CD4 (clone: GK1.5, Thermo Fisher Scientific), PE-conjugated rat anti-mouse CD71 (clone: RI7217, BioLegend), and Alexa647-conjugated rat anti-mouse CD98 (clone: RL388, BioLegend) followed by staining of the dead cell population with Fixable Viability Dye eFluor 780 (Thermo Fisher Scientific). In another cell culture, harvested cells were fixed in 0.8% formaldehyde (Sigma-Aldrich) PBS solution for 15 min at room temperature and then permeabilized using ice-cold 99.9% HPLC-grade methanol (Fisher Scientific) for 30 min on ice. To exclude dead cells, Fixable Viability Dye eFluor 780 (eBioscience) was added between the cell fixation and permeabilization steps. Intracellular phosphorylated proteins were then stained with Alexa-Fluor-488–conjugated anti–phospho-S6 ribosomal protein (S235/S236) (clone: D57.2.2E, Cell Signaling Technology) and APC-conjugated anti-phospho AKT (S473) (clone: SDRNR, eBioscience) for 30 min at 4°C. Cell-surface and intracellular phosphorylated protein were analyzed on LSRFortessa (Becton Dickenson).

### Cytokine Secretion From Mouse T Cell Subsets Stimulated *in vitro*

Splenic CD4 or CD8 T cells from prediabetic NOD mice were purified using either CD4 or CD8 microbeads (Miltenyi Biotecs) or FACS-sorted using a FACSAria II (Becton Dickinson) into CD62L^high^CD44^low^ (naïve), CD62L^high^CD44^high^ (central memory, CM), or CD62L^low^CD44^high^ (effector memory, EM) CD4 or CD8 T cell subsets using PE-conjugated rat anti-mouse CD62L (clone: MEL-14, BD Biosciences), Alexa-Fluor-700–conjugated rat anti-mouse CD44 (clone: IM7, BD Biosciences), FITC-conjugated rat anti-mouse CD4 (clone: GK1.5, BioLegend), and APC-conjugated rat anti-mouse CD3e (clone: 17A2, BioLegend) for CD4 T cell subset sorting, or BD Horizon V500-conjugated rat anti-mouse CD8 (clone: 53-6.7, BD Biosciences) and FITC-conjugated hamster anti-mouse TCRβ (clone: H57-597, BD Biosciences) for CD8 T cell subset sorting; 1 × 10^5^ cells of each sorted cell subset were resuspended in 0.2 mL of TCM as described above and stimulated with 25,000 beads/well of CD3/CD28 dynabeads (Invitrogen) for 24 h in the presence or absence of recombinant sCD137 on a 96-well round-bottom plate. In some experiments, 1 × 10^5^ cells of magnetically purified CD4 or CD8 T cells were stimulated with 25,000 CD3/28 dynabeads for a total of 72 h, and sCD137 was added at indicated time points. IL-2 and IFN-ɤ protein concentrations in collected supernatants were measured by ELISA using IL-2 ELISA ready-set-Go! (eBioscience) and IFN-ɤ ELISA ready-set-Go! (eBioscience), respectively, following the manufacturer's instruction.

### Cell Cycle Analysis

Splenic CD4+ T cells were magnetically purified and stimulated as described above, with or without sCD137 for 48 h. The percentages of harvested CD4 T cells in G0/G1, S, or G2/M phase were analyzed using FITC BrdU Flow kit (BD Biosciences), following the manufacturer's protocol. Briefly, 10 μM (final concentration) of BrdU was added 45 min before harvesting, fixing, and permeabilizing cells. Cells were incubated for 1 h in the presence of 300 μg/mL of DNase-PBS solution at 37°C and stained with anti-BrdU FITC antibody and 7-AAD. The percentage of cells in each cell cycle phase was then evaluated on FACSCalibur (BD Biosciences).

### Gene Expression Analysis

CD44-positive or -negative (naïve) CD4 or CD8 T cells were purified using CD4 or CD8 naïve T cell isolation kit (Miltenyi Biotec). Fifty thousand CD44-positive or CD44-negative (naïve) cells resuspended in 0.2 ml/well of TCM were stimulated with 50,000 CD3/28 beads and harvested at the indicated times in 96-well round-bottom plate. Messenger RNA was extracted from harvested cells using RNeasy Plus Mini Kits (QIAGEN). cDNA was synthesized by PrimeScript RT Master Mix (Takara, Inc.), and semi-quantitative PCR was performed using SYBR® FAST qPCR Master mix and other KAPA reagents (KAPA Biosystems, Inc.). All qRT-PCR experiments were performed in duplicate on a StepOnePlus Real-time PCR system (Applied Biosystems). *Tnfsf9* (CD137L) gene expression data were normalized to *Gapdh* expression. Mm_Gapdh_3_SG and Mm_Tnfsf9_1_SG QuantiTect Primer Assay (Qiagen) for *gapdh* and *Tnfsf9* gene expression, respectively, were used as primers.

### Induction and Restimulation of Differentiated Th1 Cells *in vitro*

Splenic CD4 T cells (1 × 10^6^) isolated from 10-week-old prediabetic female NOD mice were stimulated with plate-bound anti-CD3 antibody (3 μg/ml, clone: 145-2C11, BD Biosciences, USA) and soluble anti-CD28 antibody (3 μg/ml, clone: 37.51, BD Biosciences) in the presence of recombinant mouse IL-12 (10 ng/mL, TONBO Biosciences), recombinant mouse IL-2 (5 ng/mL, Invitrogen, USA), and anti-IL-4 antibody (10 μg/ml, clone: 11B11, BD Biosciences, USA) in 12-well culture plates. After 5 days, cells were harvested and washed, and 1 × 10^6^ differentiated Th1 cells were treated with plate-bound anti-CD3 antibody (1 μg/ml) and soluble anti-CD28 antibody (1 μg/ml) in the presence or absence of recombinant sCD137 (240 μg/mL) for 24 h. In some wells, recombinant mouse IL-2 (25 U/ml) was added. After 24 h, cells were washed and rested in fresh TCM without IL-2 for 48 h; 0.5 × 10^5^ rested living cells were restimulated with plate-bound anti-CD3 antibody (0.5 μg/ml) and soluble anti-CD28 antibody (0.5 μg/ml) for 24 h. Supernatant was collected, and IL-2 concentration was measured by ELISA.

### Production of CD137L-Expressing Cells Lines and Testing for Specific sCD137 Binding

(1) Construction of CD137L-expressing cell lines. TrueORF cDNA expression clones for C-terminal Myc- and DDK (Flag)–tagged *M. musculus* TNFSF9 were obtained (cat. # MR220933, Origen). Circular plasmid DNA was isolated and restricted with ApaLI (NEB) to linearize the vector DNA. Linearized DNA was then transfected into HEK293 cells using Lipofectamine 2000 and stable integrants selected for using 400–500 μg/ml of Geneticin (G418). After isolation and expansion of individual cell clones, high mouse CD137L-Myc-DDK expressers were stained with rat anti-mouse CD137L antibody (clone: TKS-1, BioLegend) or Rat IgG2a isotype control antibody (clone: RTK2758, BioLegend), followed by biotinylated mouse anti-Rat IgG2a antibody (clone: RG7/1.30, BD Biosciences) and streptavidin PE (BioLegend), identified using BD FACSCanto (BD Biosciences).

(2) Confirmation of specific binding of sCD137 to CD137L. HEK-mCD137L or control HEK293 cells (2 × 10^6^) were preincubated with 1 ug/sample of anti-CD137L Ab (TKS-1) or the same volume of PBS for 30 min at 4°C. After washing cells to remove excess CD137L antibody, cells were incubated with titrated doses of sCD137 for 30 min at 4°C. Then cells were stained with biotinylated rat anti-mouse CD137 antibodies (clone: 17B5, BioLegend) followed by streptavidin PE (BioLegend). The level of bound CD137 was analyzed using FACSCanto (BD Bioscience).

### Analysis of Human PBMC and Serum Samples for sCD137

Unused residual peripheral blood samples collected for routine clinical labs from pediatric T1D patients and age-matched unrelated controls (all patients between ages 2 and 18 years) were de-identified by clinical lab staff at Cincinnati Children's Hospital. The diabetic patients were equally split between new-onset and established patients; mean duration of disease in established patients was 6.7 years. Notably, however, all diabetic patients were admitted for acute T1D disease flares. Serum/plasma was tested for human sCD137 level by ELISA using the Human 4-1BB/TNFRSF9 DuoSet ELISA kit (R&D Systems, Minneapolis, MN), following the manufacturer's instructions.

Peripheral blood mononuclear cells from healthy donors were separated by centrifugation through Ficoll-Hypaque (GE, Fairfield, CT). Resting CD4+ T cells were purified by negative selection using a CD4+ T cell isolation kit (Miltenyi Biotec, Auburn, CA), following the manufacturer's instructions. To isolate CD4 T cell subsets, purified CD4+ T cells were stained with FITC-conjugated mouse anti-human CD8 (clone: RPA-T8, eBioscience), PE-conjugated anti-human CD25 (clone: REA945, Mitenyi Biotec), and APC-conjugated mouse anti-human CD127 (eBioRDR5, eBioscience), and sorted using a FACSAria (BD Biosciences). The subsets were defined as follows: Tregs (CD8–CD25^hi^CD127^lo^ cells), double positive T cells (Tdps, CD8–CD25^hi^CD127^hi^), and conventional T cells (Tconvs, CD8–CD25^lo^CD127^hi^). Purity of the sorted populations was >90%, as determined by post-sorting analysis of FOXP3 expression. Fifty thousand Treg, Tdp, and Tconv cells were separately cultured on a 96-well round-bottom cell culture plate with human recombinant IL-2 at indicated doses. In some wells, 50,000 CD3/28 beads were added. Cells were cultured for 72 h, and supernatant was collected. Human sCD137 protein concentration was measured by ELISA using the Human 4-1BB/TNFRSF9 DuoSet ELISA kit (R&D Systems, Minneapolis, MN) following the manufacturer's instructions.

### Cell Proliferation Assay of Human CD4 T Cells With Human sCD137

Human CD4 T cells were sorted from human PBMCs by the method described above and stained with anti-human CD8-FITC (clone: RPA-T8, eBioscience), anti-human CD25-APC (clone: REA945, Miltenyi Biotec), and anti-human CD127-PE-Cy7 (R34.34, Beckman Coulter), and sorted using an SH800S cell sorter (Sony biotechnology). Tconv cells were labeled with carboxyfluorescein succinimidyl ester (CFSE, Invitrogen) at 0.5 μM final concentration. Fifty thousand CFSE-labeled Tconv cells were cultured on a 96-well flat-bottom cell culture plates with 50,000 human CD3/28 dynabeads (Thermo Fisher Scientific) in the presence or absence of 40 μg/ml of human 4-1BB Fc chimera protein (R&D Systems) for 72 h. CFSE dilution of harvested cells was analyzed on FACSCanto (BD Biosciences).

### Data Analysis

All data acquired in flow cytometry were analyzed using FlowJo software version 10 (FlowJo, LLC). All statistical analysis was performed using GraphPad Prism version 6 for Windows (GraphPad software). Significance testing was done using either the unpaired *t*-test or Mann–Whitney tests for sample comparisons, ANOVA and *post hoc* analysis in continuing parametric data, and the log-rank test for survival curve analysis. Results in bar graphs are expressed as the mean ± standard error of the mean. *P*-values < 0.05 were considered statistically significant.

## Results

### sCD137 Treatment Ameliorates New-Onset Diabetes in NOD Mice and Can Preserve Insulin-Expressing Islet Beta Cells

**Figure 1:**
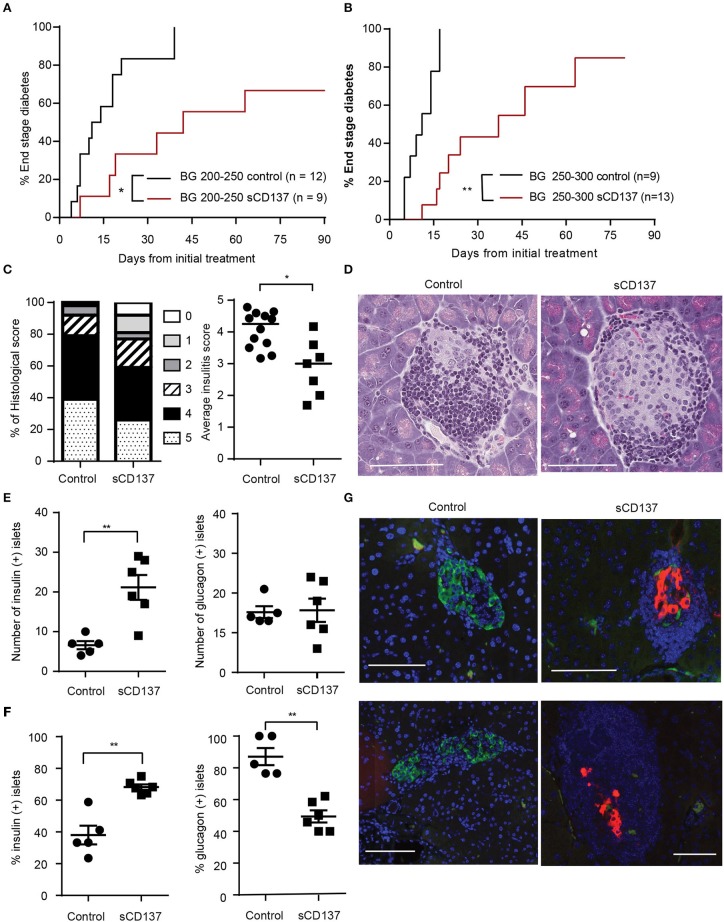
Soluble CD137 (sCD137) ameliorates acute type 1 diabetes (T1D) in nonobese diabetic (NOD) mice. **(A,B)** Survival curves of acutely diabetic mice treated with sCD137 vs. controls. Female NOD mice were monitored until they became diabetic [blood glucose of **(A)** 200–250 mg/dl or **(B)** 250–300 mg/dl], and were then treated with sCD137 vs. untreated **(A)** or treated with PBS **(B)** (see methods). ^*^p < 0.05, ^**^p < 0.01, Mantel–Cox log-rank test. **(C)** Insulitis severity (see methods) in sCD137-treated mice (*n* = 7) that did not progress to end-stage T1D and control mice (*n* = 12) with end-stage diabetes. ^*^p < 0.05, Mann–Whitney *U*-test. **(D)** Representative H&E-stained islets from each group. **(E–G)** The pancreatic islet was stained for insulin (red), glucagon (green), and nucleus (blue) and imaged by dual immunofluorescence in sCD137-treated mice (*n* = 6) compared to PBS-treated, end-stage diabetic mice (*n* = 5). The number **(E)** and percentages **(F)** of insulin- (**E,F**, left) and glucagon- (**E,F**, right) positive islets per section are shown. ^**^p < 0.01, Mann–Whitney *U*-test **(E)** and unpaired *t*-test **(F)**. **(G)** Representative islets from two independent sCD137- vs. PBS-treated mice showing insulin preservation in sCD137-treated mice. White bars = 100 μm.

Halting acute T1D remains an unachieved goal, and new therapeutic approaches are needed. Although we previously published that we can prevent T1D in NOD mice using sCD137 (11), this is not a good indicator of the ability of sCD137 to treat an acute autoimmune disease. Many agents that have “prevented” T1D in NOD, such as LPS, failed to reverse the acute disease (15). Therefore, we tested the effect of sCD137 on acute T1D. In the first treatment group, we started treatment with sCD137 in NOD mice with initial blood glucose between 200 and 250 mg/dl and showed a delay in progression to end-stage (BG >500 mg/dl) diabetes; in addition, three mice did not progress to end-stage disease (Figure 1A). In the second treatment group, we chose the more challenging criteria of initial BG between 250 and 300 mg/dl. PBS-treated control mice showed a more rapid progression to end-stage disease; the sCD137-treated mice had significantly delayed disease progression overall, and four mice did not progress to end-stage disease (Figure 1B). The individual blood glucose measurements at each time point are shown in Supplemental Figure 1A; clearly, all sCD137-treated mice (curves shown in red) are shifted to the right compared to the control mice (black curves). A direct ameliorative effect of sCD137 is shown by comparing the effects of sCD137 to PBS or no treatment on the next blood glucose measurement. As shown in Supplemental Figure 1B, in the seven mice that did not progress to end-stage T1D, sCD137 treatment on average reduced the next BG measurement by 22.1 mg/dl, a statistically significant difference compared to a mean blood glucose increase of 73.2 mg/dl in all the control mice (Supplemental Figure 1B). The mean ending BG level was >500 mg/dl in all control mice, compared with a mean of 213.9 mg/dl in the seven mice that did not progress (whose mean initial BG prior to treatment was 232.9 mg/dl). Histologically, successfully treated mice had a significantly decreased insulitis score, with decreased numbers of severely affected islets (Figures 1C,D). Beta cell immunohistochemistry demonstrated significant preservation of insulin+ beta cells, and an increase of insulin-positive islets as a percentage of all islets, in successfully treated mice (Figures 1E–G). The number of glucagon-positive islets was not statistically different between the two groups, but the percentage of glucagon-positive islets decreased in the sCD137-treated mice (Figures 1E–G, see discussion). Overall, treatment of new-onset diabetic NOD mice with sCD137 significantly delayed development of end-stage diabetes (blood glucose >500 mg/dl) compared with controls, and several mice did not progress to end-stage disease while being treated. These data indicate that sCD137 has a therapeutic potential in acute, new-onset autoimmune diabetes.

### sCD137 Induces T Cell Anergy; Exogenous IL-2 Prevents and Reverses sCD137 Suppressive Effect on Antigen-Specific T Cell Proliferation and Cytokine Production

We previously showed that sCD137 can suppress both CD4+ (11) and CD8+ (12) T cell proliferation and that the suppressive effect of recombinant sCD137 could be blocked by an anti-CD137L antibody (TKS-1), but this did not directly show ligand specificity (11). We constructed a CD137L-expressing cell line (in a human cell line that does not express mouse CD137L) and showed that it expressed high levels of recombinant CD137L (Supplemental Figure 2A). Next, using a previously published approach (16), we showed that sCD137 specifically bound the recombinant CD137L (Supplemental Figure 2B) and that this binding could be blocked with the TKS-1 anti-CD137L antibody (Supplemental Figure 2C). These results confirmed the specificity of our sCD137 reagent.

**Figure 2:**
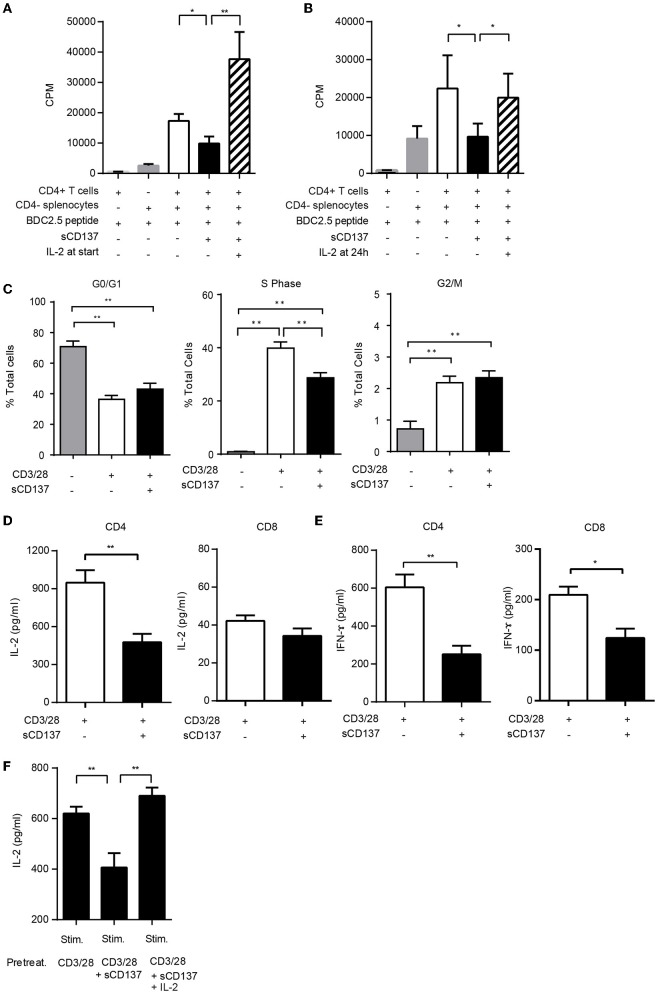
sCD137 induces T cell anergy: IL-2 prevents and reverses sCD137 suppressive effect on antigen-specific T cell activation. **(A,B)** CD4+CD25– T cells and CD4-negative splenic antigen-presenting cells purified from non-diabetic NOD BDC2.5 transgenic mice were co-cultured with BDC2.5 peptide in the presence or absence of sCD137. In some wells, 25 U/ml of recombinant mouse IL-2 was added at the start **(A)** or 24 h **(B)** after starting cell culture. Cell proliferation was counted on a β-scintillation counter. Data expressed as mean/SEM from *n* = 3 **(A)** and *n* = 5 **(B)** independent samples in three independent experiments. ^*^p < 0.05, ^**^p < 0.01, unpaired *t*-test. **(C)** Magnetically isolated CD4 T cells were cultured with CD3/CD28 beads and sCD137 for 48 h, and cell cycle phases were analyzed by flow cytometry. Data expressed as mean/SEM, *n* = 4 mice. ^*^p < 0.05, ^**^p < 0.01, Mann–Whitney *U*-test. **(D,E)** MACS-bead-purified splenic NOD CD4+CD25– or CD8 T cells were stimulated with CD3/28 in the presence/absence of sCD137 for 24 h. After 24 h, supernatant was collected, and IL-2 **(D)** and IFN-ɤ **(E)** concentration was measured by ELISA. Data expressed as mean/SEM from three biologically independent experimental samples. **(F)** Differentiated Th1 cells (1 × 10^6^) were stimulated for 24 h with CD3/28 beads, in the presence and absence of sCD137, with or without IL-2. Cells were then washed and rested in fresh cell culture media without IL-2 for 48 h, and then 0.5 × 10^5^ rested living cells were restimulated for 24 h with plate-bound aCD3Ab (0.5 μg/ml) and soluble CD28 Ab (0.5 μg/ml). IL-2 in supernatant was measured by ELISA. Data expressed as mean and standard errors of mean from three independent experiments. ^**^p < 0.01, unpaired *t*-test.

The mechanism of the T cell suppressive effect was unclear, so we tested whether sCD137-treated T cells showed an anergic phenotype in antigen-specific CD4+ T cell proliferation assays, using T cells from NOD BDC2.5 congenic mice. Exogenous IL-2 added at the start of the T cell culture prevented sCD137-mediated antigen-specific T cell proliferation (Figure 2A). To meet the criteria for T cell anergy, however, the established, suppressed T cell phenotype must be reversible by IL-2. We tested the effect of adding IL-2 after induction of suppression and showed that IL-2 added 24 h after cell culture reversed the antigen-specific sCD137 suppressive effect on BDC 2.5 CD4+ transgenic T cells (Figure 2B). Consistent with Figures 2A,B, sCD137 suppressed the S phase of the cell cycle (Figure 2C). Cytokine secretion including IL-2 (Figure 2D) from CD3/28-stimulated CD4 and IFN-ɤ (Figure 2E) from CD3/28-stimulated CD4 and CD8 T cells was also suppressed by sCD137 (Figure 2E). To test whether the cytokine reduction induced by sCD137 was a reversible functional component of T cell anergy, we differentiated CD4 T cells into Th1 cells *in vitro*, restimulated them using CD3/28 plus/minus sCD137 and/or IL-2, washed them, and then rested them for 48 h in fresh TCM without IL-2 prior to restimulation. The reactivated Th1 cells that had been treated with sCD137 produced less IL-2 after restimulation, and this suppression of cytokine production was reversed by IL-2 treatment prior to restimulation (see methods) (Figure 2F). This result indicated that sCD137 treatment induces an anergic phenotype in T cells through its direct effect on T cells and that sCD137 could suppress antigen-experienced, cytokine differentiated cells and activated memory T cells, suggesting the ability to ameliorate ongoing autoimmunity against beta cells.

### sCD137 Downregulates T Cells via the mTORC1 Signaling Pathway

Little is known about the CD137L pathway signaling in T cells, but in antigen-presenting cells, existing data suggest that membrane CD137/CD137L interaction acts via downstream Akt and mTOR pathways (17). Therefore, we hypothesized that sCD137 caused decreased CD4/CD8 T cell proliferation via the AKT and/or mTOR pathways. In mammalian cells, mTOR forms two enzyme complexes, mTOR complex 1 (mTORC1), and mTOR complex 2 (mTORC2). We evaluated sCD137's effect on the mTOR pathway in T cells using phosphorylated S6 ribosomal protein at S235/236 as a marker of mTORC1 activity and phosphorylation of AKT at S473 as a maker of mTORC2 activity.

**Figure 3:**
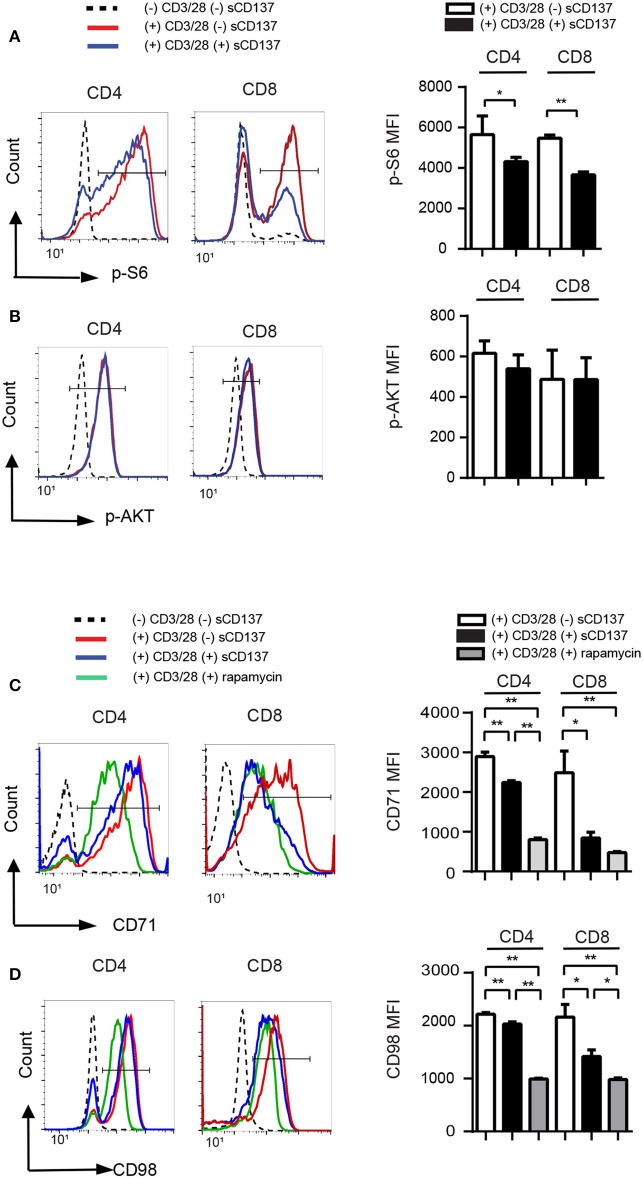
sCD137-mediated suppression associated with reduced mTORC1 pathway activation; no effect on Akt pathway. NOD CD4+CD25– or CD8 T cells were stimulated with CD3/28 beads in the presence of sCD137 or rapamycin for 24 h. After 24 h culture, cells were harvested, and intracellular or cell-surface proteins were stained and analyzed as described in the *Materials and Methods* section. One representative histogram of intracellular p-S6 **(A)**, p-AKT **(B)**, cell-surface CD71 **(C)**, and CD98 **(D)** expression in CD4+CD25– and CD8+ cells is shown. Geometric MFI (far right) of the indicated populations is shown as mean/SEM from three to four biologically independent experimental samples. ^*^p < 0.05, ^**^p < 0.01, unpaired *t*-test.

Phospho-flow cytometry showed that sCD137 significantly reduced p-S6 MFI in both CD4+ and CD8+ T cells (Figure 3A), while p-AKT (S473) expression did not change significantly (Figure 3B). The percentage of p-S6+ cells, but not p-AKT+ cells, was also significantly decreased in the sCD137-treated group (not shown). CD71 is a transferrin transporter that plays a crucial role in DNA synthesis in lymphocytes (18, 19), and CD98 is an amino acid transporter that is essential for cell expansion (20); both are regulated by mTORC1 activity in T cells (21, 22). sCD137 treatment significantly reduced CD71 and CD98 MFI in both CD4 and CD8 T cells, with a greater overall reduction in CD8 cells (Figures 3C,D). We compared these effects to the known mTORC1 inhibitor, rapamycin; sCD137 suppressed the expression of these molecules comparably to rapamycin in CD8 cells, with somewhat less suppression in CD4 cells (Figures 3C,D). We also directly compared sCD137 cytokine suppression of IFN-γ and IL-2 to rapamycin; both agents suppressed these cytokines with similar efficacy (Supplemental Figure 3). These results indicated that sCD137/CD137L interaction reduced mTORC1 activity, resulting in downregulation of T cell metabolic transporter expression and anergy induction.

### sCD137 Acts on T Cells Early in the Cell Cycle, Related to Kinetics of CD137L Upregulation; Differential Effects of sCD137 on T Cell Memory Subsets

**Figure 4:**
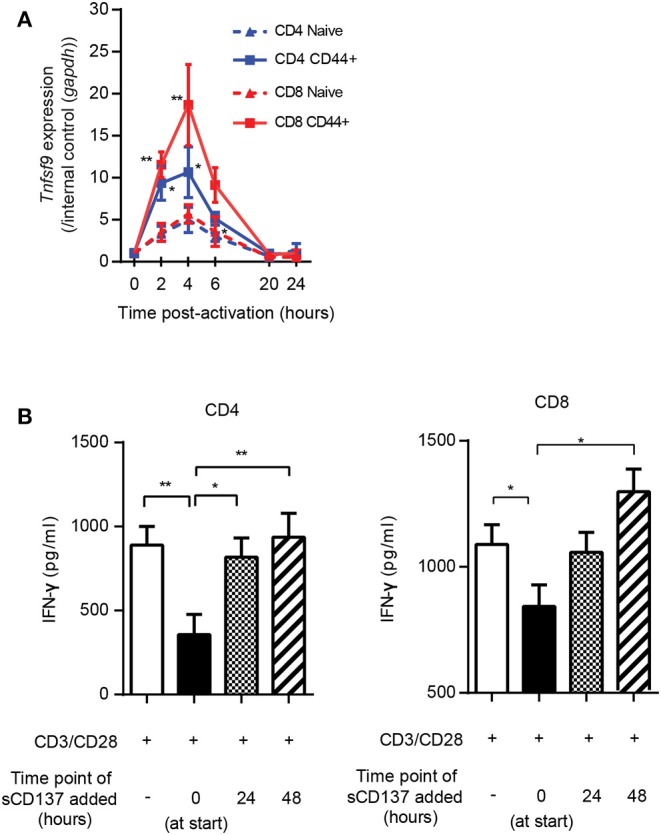
Kinetics of CD137L upregulation; sCD137 suppresses T cell cytokine production only when administered within 24 h of T cell activation. **(A)** The kinetic profile of *Tnfsf9* gene expression in naïve (CD44–) and memory (CD44+) T cells stimulated with CD3/28 beads from three independent experiments. RT-PCR data were normalized based on the expression in each subset at 0 h (baseline). ^*^p < 0.05 vs. naïve CD4, ^**^p < 0.05 vs. naïve CD8, ANOVA. **(B)** NOD CD4+ or CD8+ T cells were stimulated with CD3/CD28 dynabeads and cultured with sCD137 added at indicated time points. After a total of 72 h culture, supernatant was collected and IFN-ɤ concentration measured by ELISA. Data expressed as mean and standard errors of mean from three biologically independent experiments and analyzed by ANOVA. ^*^p < 0.05, ^**^p < 0.01.

The studies in Figure 3 showed a larger effect of sCD137 on CD8 T cell p-S6, CD71, and CD98 downregulation compared to CD4 cells. To understand this, we looked at the kinetics of CD137L expression on CD4 and CD8 T cell subsets. CD137L protein is difficult to detect on the T cell surface due to formation of cis-complexes with CD137 (23, 24) and possible limitations of the available antibodies (24), so we assessed *Tnfsf9* (encoding CD137L) gene expression using real-time PCR. *Tnfsf9* expression peaked 4 h after stimulation (Figure 4A), and *Tnfsf9* expression was significantly higher in CD44+ (activated/memory) T cells. CD8 memory/activated cells showed a significantly greater upregulation of *Tnfsf9* than CD4 cells after stimulation *in vitro* (Figure 4A). The early upregulation of CD137L suggested that sCD137 would act early after T cell activation but might be less effective later. To test this, we added sCD137 to CD4 and CD8 T cell cultures at different time points after CD3/CD28 stimulation and measured the cytokine production total 72 h post–CD3/28 stimulation. As in Figure 2, sCD137 added at the start of cell cultures significantly reduced IFN-ɤ (Figure 4B) concentration, but sCD137 added at either 24 or 48 h did not suppress CD4 or CD8 T cell cytokine production. These data confirmed that sCD137 mainly suppresses CD4 T cell cytokine production within the first 24 h after activation.

**Figure 5:**
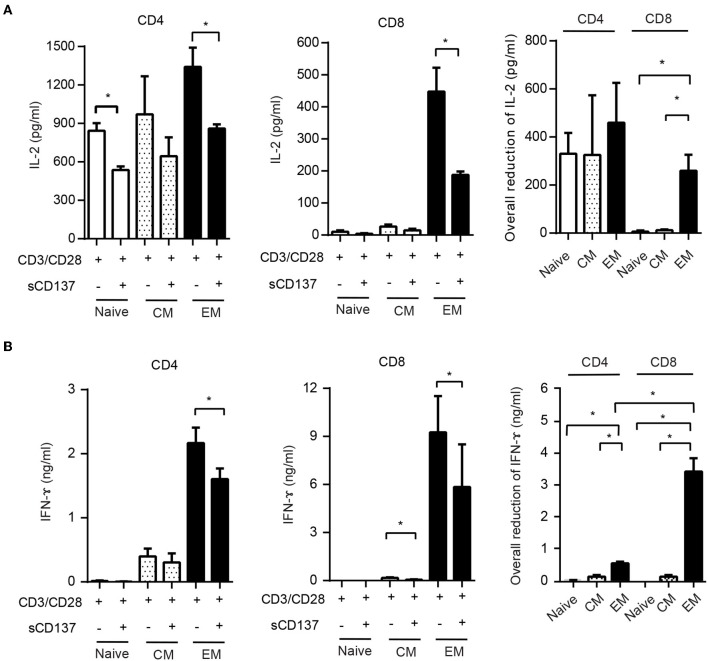
Differential effect of sCD137 on cytokine production; CD8 effector memory T cells have the greatest reduction in cytokine expression. **(A,B)** Naïve (CD62L^high^CD44^low^), central memory (CD62L^high^CD44^high^), and effector memory (CD62L^low^ CD44^high^) subsets were sorted from NOD CD4 or CD8 T cells and cultured with CD3/CD28 beads and sCD137 for 24 h. IL-2 **(A)** and IFN-ɤ **(B)** concentrations were measured by ELISA. Overall reduction of IL-2 or IFN-ɤ was calculated by subtraction of total cytokine in the sCD137-treated group from the cytokine level in the CD3/28 group in each individual experiment (*n* = 3 experiments). ^*^p < 0.05, ^**^p < 0.01, unpaired *t*-test. CM, central memory subset; EM, effector memory subset.

This result led us to study the functional effects of sCD137 in FACS-sorted CD4 and CD8 cell subsets. We sorted naïve (CD62L^high^CD44^low^), CM (CD62L^high^CD44^high^), and EM (CD62L^low^CD44^high^) subsets from splenic CD4 or CD8 T cells, stimulated with CD3/CD28 beads for 24 h, and assayed IL-2/IFN-ɤ expression. sCD137 reduced CD4 IL-2 concentration in all three subsets (Figure 5A, left panel). In CD8 subsets, only EM cells produced substantial amounts of IL-2 (Figure 5A, middle panel), and sCD137 significantly reduced IL-2 in EM CD8 cells (Figure 5A, right panel). In contrast, CD4 and CD8 EM T cells were the major source of IFN-ɤ, and sCD137 exerted most of its suppression of IFN-ɤ production in this subset (Figure 5B, right and middle panel, respectively). Overall, the vast majority of IFN-γ reduction resulted from sCD137 action on the CD8 EM subset (Figure 5B, right panel). These data indicate that upregulation of CD137L in memory cells predisposes these cells, and especially CD8 EM cells, to suppression by sCD137, resulting in significant downregulation of inflammatory cytokine expression, which plays a major role in T1D.

### Human sCD137 Is Decreased in Type 1 Diabetic Patients, Is Secreted From Tregs, and Suppresses Human T Cells

**Figure 6:**
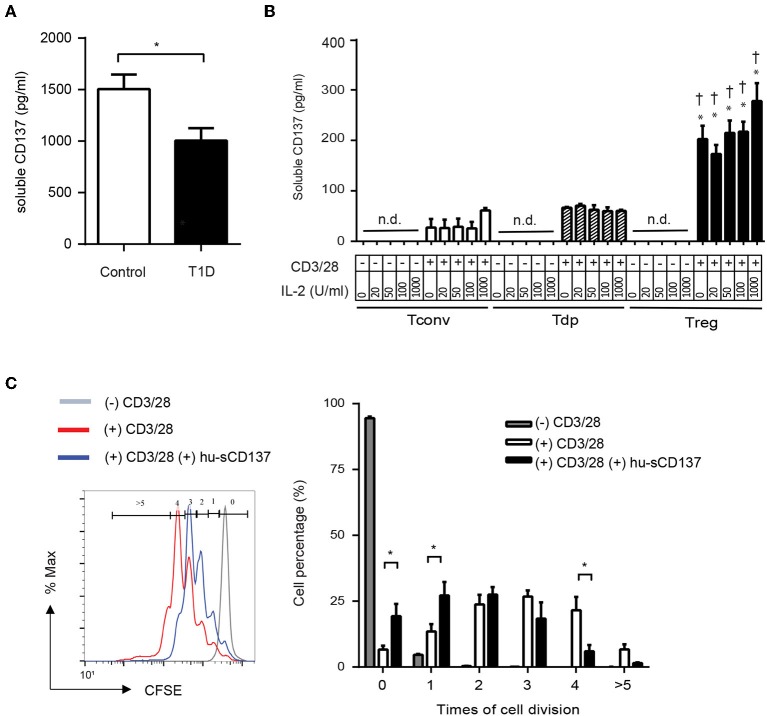
Human T1D patients have decreased serum sCD137 levels, and sCD137 is secreted from human regulatory T cells. Human sCD137 suppresses human T cell proliferation. **(A)** sCD137 concentration in serum from pediatric type 1 diabetic patients (*n* = 11) or age-matched controls without T1D (*n* = 10) (all subjects were ages 2–18 years) was measured by ELISA. ^*^p = 0.03, unpaired *t*-test. **(B)** Human CD4+CD25^low^CD127^high^ conventional T cells (Tconvs), CD4+CD25^high^CD127^high^ double positive T cells (Tdps), and CD4+CD25^high^CD127^low^ regulatory T cells (Tregs) from healthy donors were stimulated as indicated for 72 h. Supernatant was collected and human sCD137 concentration measured by ELISA. Data were expressed as mean/SEM from four subjects. ^*^p < 0.01 vs. Tconv in each dose of IL-2, ^†^*p* < 0.01 vs. Tdp in each dose of IL-2, unpaired *t*-test. n.d., not detected. **(C)** carboxyfluorescein succinimidyl ester (CFSE)–labeled human CD4+ Tconv cells were stimulated with CD3/28 dynabeads in the presence or absence of hu-sCD137 for 3 days. CFSE dilution was analyzed on FACSCanto. One representative histogram (left panel) of CFSE is shown. Percentages of cells with the indicated number of cell divisions (gated as shown in the histogram) are shown in the right bar graph. Data expressed as average/SEM of *n* = 5 independent samples ^*^p < 0.01, unpaired *t*-test.

Finally, we investigated whether human T1D patients showed decreased serum sCD137 compared to controls, analogous to NOD vs. disease-resistant NOD *Idd9.3* mice (10). Serum sCD137 was significantly lower in pediatric type 1 diabetic patients admitted with acute disease exacerbations compared to age-matched non-diabetic donors (Figure 6A). Next, we determined whether human Tregs were the primary source of sCD137, as we found in mice (10). CD4+CD25^high^CD127^low^ Tregs FACS-sorted from peripheral blood mononuclear cells secreted human sCD137, but CD4+CD25^low^CD127^high^ Tconvs, and CD4+CD25^high^CD127^high^ (Tdp) cells did not (Figure 6B). These data support the hypothesis that insufficient levels of anti-inflammatory sCD137 secreted from Tregs might contribute to the progression/exacerbation of human T1D. Finally, we tested whether human sCD137 could suppress human T cells. Using a human Fc-CD137 protein, human peripheral CD4 cell proliferation showed significant suppression when stimulated in the presence of sCD137 compared to CD3/CD28 alone (Figure 6C), very similar to our results in mice (11). These results suggest that the biology of sCD137 production and its biological effects to suppress T cells are very similar in humans compared to mice and support further investigation of sCD137 as a therapeutic agent in T cell–mediated human autoimmune disease.

## Discussion

T1D remains an incurable disease, despite much elucidation of its immunopathological basis in the last five decades. T cells are clearly central to the pathogenesis of the disease, yet antigen-specific T cell therapy has failed in over 20 human clinical trials (15). Clearly, ongoing investigation of T1D immunopathogenesis and development of new therapeutic approaches (including synergistic combination therapies) are both needed. We have focused on a genetically linked immune pathway in NOD mice: the *Tnfsf9* gene (encoding CD137) is most likely the causative B10 protective allele in the *Idd9.3* region and encodes a critical immune regulatory molecule that functions both on the cell surface and in an alternatively spliced, secreted form (sCD137). In this paper, we present strong evidence that sCD137 induces T cell anergy, sufficiently robust to ameliorate acute T1D. Disease amelioration shown here included a significant delay in disease progression, lack of progression to end-stage disease in some sCD137-treated mice (but in none of the controls), preservation of insulin-positive islets, decreased overall islet inflammation, and significant evidence of a therapeutic effect of sCD137 during flares of disease (Supplemental Figure 1B). In addition, we saw evidence of insulin-mediated paracrine suppression of glucagon in the islets (see below). Nonetheless, many sCD137-treated mice still progressed to end-stage disease, and evidence of disease activity continued in treated mice—this is clearly not a “cure” of T1D. This is perhaps an expected result, since sCD137 exclusively targets the immune pathogenesis of T1D, and there is no obvious means by which it can enhance beta cell regeneration. Hence, sCD137 would likely need to be used as a “combination” therapy in T1D, the need for which is increasingly appreciated in the field (25). Nonetheless, as a powerful new immunotherapeutic approach, these results support further investigation of sCD137 as a T cell directed therapy that could treat human T1D.

Little is known about CD137L signaling in T cells, compared to at least some studies in APCs. Partly this is a result of the difficulty in studying CD137L expression at the protein level; it is hard to detect, since it often complexes in *cis* with CD137 and is endocytosed (24). We overcame that issue by expressing CD137L in a non-expressing cell line and demonstrated specific binding of our recombinant protein to CD137L (Supplemental Figure 2). T cell anergy is probably best described as clonal unresponsiveness reversible by IL-2, as originally described by Schwartz (26), and our results meet that definition (Figure 2B). The mechanisms of anergy induction are complex and still incompletely understood; moreover, there are many forms of T cell unresponsiveness and several different anergy models (27). We show that CD4+ T cells treated with sCD137 have decreased p-S6, which is a downstream molecule in the mTORC1 pathway, while no effect is seen on pAKT, which is a downstream target in the mTORC2 pathway (Figure 3B) (28). In addition, we see decreased expression of CD71 and CD98, which are mTORC1-dependent proteins involved in DNA synthesis and cell proliferation. These results identify the mTORC1 pathway as a crucial aspect of sCD137-mediated unresponsiveness. Notably, we did not demonstrate antigen-specific CD8 T cell anergy, which should be done in future studies. We show that sCD137 has comparable effects on the expression of CD71 and CD98 (mTORC1 markers) as the mTORC1 specific immunomodulator rapamycin; especially in CD8 cells. This raises the interesting question as to whether sCD137 could function as an endogenous rapamycin equivalent. This comparison and hypothesis, however, will have to be explored in future studies, which could also be extended to include *in vivo* effects of sCD137 on circulating and islet-infiltrating T cells.

We show that the EM CD8 T cell subset has the most marked reduction of IFN-γ production by sCD137 (Figure 5B); in addition, CD8 T cells show significantly more sCD137-mediated downregulation of p-S6, CD71, and CD98 in the mTORC1 pathway than CD4 cells (Figures 3C,D). A large amount of literature has documented the importance of CD8 T cells in T1D pathogenesis over the last three decades (29–31). The current consensus is that CD8 T cells are the ultimate mediator of beta cell destruction (32), most likely via perforin and Fas mediated pathways (33, 34). Of particular note is the critical role of EM CD8 T cells in the final phase of beta cell destruction; T EM CD8 cells are now well-recognized as a prime target for emergent T1D therapies (35, 36). Most strikingly, Herold et al. have recently used a T cell library approach to show that activated, memory CD8 T cells expressing IFN-γ are critical cell types in T1D patients that can now be tracked *in vivo* (37). These findings highlight the importance of our finding that sCD137 primarily targets EM CD8 T cells to dramatically lower IFN-γ, and support further investigation of this approach as a treatment for human T1D.

A central issue is understanding how sCD137 functions to suppress T cell activation. Membrane CD137 can induce activation signals in T cells, while the sCD137 form induces anergy. It has recently been demonstrated that the affinity of TNF/TNFR binding is largely determined by the number of oligomers in the complex (38). As a result, two key observations may explain the difference between membrane and sCD137 signaling. First, we demonstrated previously that sCD137 exists as a dimer, not as a trimer as was previously reported (11). The interaction of a dimer with its ligand decreases the number of stimulating protein subunits (compared to a trimer) and may also completely change the geometry of receptor–ligand complex formation. Second, Croft et al. have recently reported that CD137L also exists as a dimer, not as a trimer as previously reported (39). This finding may completely change our understanding of CD137/CD137L biology and hence the differential effects of membrane vs. soluble forms of CD137.

We note some additional effects of sCD137 that go beyond its effects on T cell biology. First, in Figures 1E–G, we made the initially puzzling observations that while the number of glucagon+ islets in the sCD137-treated mice did not decrease, the percentage did: in other words, the increased number of insulin+ islets in treated mice didn't raise the percentage of glucagon+ islets. Inspection of individual islets confirmed (Figure 1G) that many insulin+ islets in the treated mice showed suppression of glucagon. This finding highlights an increasing awareness of the role of hyperglucagon production in acute T1D (40, 41). Physiologically, insulin suppresses glucagon secretion from alpha cells in the islets in a paracrine manner. Insulin preservation in the treated islets, therefore, likely has a secondary effect of intra-islet glucagon suppression, reflected in a decrease in glucagon+ insulin+ islets. Overall, both insulin preservation and glucagon suppression might contribute to amelioration of T1D, as suggested by a number of papers published in recent years (40, 41). We would predict that this is not an effect specific to sCD137 treatment, however, but to our knowledge, this has not been well-studied in acute T1D in NOD and should be explored in more depth in future studies.

In addition to the effect on T cells and on local glucagon production, it is possible that sCD137 has effects on other cell types and pathways beyond T cells and anergy. Recently, the role of IDO1 on pancreatic beta cells has been described, as well as a loss of indoleamine dioxygenase in T1D (42, 43). These results highlight possible regulatory roles of APCs and other immune cells; clearly, several APCs express CD137L and could be affected by sCD137 treatment, which has not been explored here. Furthermore, just as on the one hand, checkpoint inhibitors can precipitate acute T1D (44), harnessing immune checkpoints to induce T cell exhaustion is another therapeutic approach (45). Although we show that sCD137 specifically targets CD8 T cells and CD8 memory subsets, we do not show whether it induces immune exhaustion in these cells, which should be explored in future studies.

A major question that must be addressed is the use of the NOD mouse as a model of T1D and its relevance to human T1D. The major strengths of the NOD model of T1D include (1) development of spontaneous autoimmune diabetes, (2) an MHC class II molecule structurally similar to the human T1D disposing MHC class II, and (3) autoreactive T cells and autoantibodies directed at autoantigens shared between humans and mice (46, 47). In addition, multiple non-MHC susceptibility genes are shared between mouse and human, heavily weighted toward immune pathways and immune regulation (48). A major difference, however, is that T1D is easy to prevent in mice, whereas prevention trials have failed in human T1D to date. The existing literature is highly biased toward prevention trials in NOD, compared to the much more difficult task of reversing or ameliorating acute disease. Already in 2005, a review of the NOD literature showed 443 “prevention” studies but only 23 reversal studies (49); currently, ~1,000 prevention studies have been done in NOD compared to an estimated 40 or 50 reversal studies. Clearly, the main clinical issue in T1D is reversal of acute disease, which is entirely different from, and more difficult than, prevention. In fact, most of the published agents that “prevent” T1D in NOD have no effect on the acute disease (15). Prevention studies do show *in vivo* biological effects and can be useful to identify pathways in disease; however, we believe that reversal studies have been underutilized in NOD. A strength of the current study is that it demonstrates that sCD137 is active in acute autoimmunity. We do not show efficacy in all mice. This is likely explained by heterogeneity in the immune state of acutely diabetic NOD, which has been noted recently in the literature but is mechanistically unexplained (50). More studies should be performed on the immunobiology of acute T1D in NOD mice to understand the variable efficacy of therapies utilized at this stage. In addition, further dosing studies with sCD137 in acute disease need to be performed to ensure that our dosing regimens are maximized.

A final issue is the relevance of NOD studies for human T1D, a question emphasized by the failure of most agents that were successful in NOD mice to show efficacy in human studies. Given this, it is critical to extend any mouse results into human T1D patients and identify how closely the biology in the mouse reflects human immunobiology. We previously showed that protected NOD. *Idd9.3* congenic mice had higher serum levels of sCD137 compared to NOD mice (10); clearly, a deficit in an immunosuppressive/regulatory molecule could enhance autoimmune progression/severity. Here we tested pediatric T1D patients vs. controls and found that human T1D patients had decreased sCD137. Of great interest is that all the T1D patients tested in this study were admitted for T1D disease flares. As shown in Supplemental Figure 1, sCD137 can treat disease flares; this favors the potential use of this agent in acute T1D. A key unanswered question here, however, is whether sCD137 levels drop during human T1D disease flares—to answer this, more studies are needed using serum samples from T1D patients with stable, well-controlled disease. It is possible, for example, that loss of immunoregulation, reflected in a drop of serum/tissue sCD137, could contribute to disease exacerbations. These issues and the behavior of sCD137 serum levels need to be examined in larger populations; nonetheless, our results support a role for treatment with sCD137 in human T1D. In addition, we had previously shown that murine Tregs are the major source of sCD137, and here we confirm that this is also the case in humans: T effectors produce minimal sCD137, and Tregs are the major cellular source. Finally, we show that the functional effects of human sCD137 on human T cells are highly similar to those in mice; human sCD137 suppresses human T cells, which is an essential result to support further investigation of this approach for human autoimmunity. These experiments support further investigation into whether CD137L-mediated suppression can be a novel treatment pathway for human T1D and other autoimmune diseases.

## Data Availability Statement

The datasets generated for this study are available on request to the corresponding author.

## Ethics Statement

Ethical review and approval was not required for the study on human participants in accordance with the local legislation and institutional requirements. Written informed consent for participation was not required for this study in accordance with the national legislation and the institutional requirements. The animal study was reviewed and approved by University of Cincinnati IACUC.

## Author Contributions

AI, KK, DA, KB, CC, RM, Y-GC, and WR conceived and planned the experiments. AI, LO, KK, YW, DA, KB, and SM performed the experiments. CC and LD obtained and contributed human samples. AI and WR analyzed the data and wrote the manuscript with support from DA and SM. CC, Y-GC, and WR supervised the project.

### Conflict of Interest

The authors declare that the research was conducted in the absence of any commercial or financial relationships that could be construed as a potential conflict of interest.

## Supplementary Material

Supplemental Figure 1 Soluble CD137 treats acute T1D and induces treatment-related drops in serum blood glucose. **(A)** All individual blood glucose measurements from all the treated and control mice in Figures 1A,B. Control mice from Figures 1A,B are shown by black symbols/lines while sCD137 treated mice from Figures 1A,B are shown by red symbols/lines. **(B)** sCD137 reduces next blood glucose measurement compared to PBS/no treatment. All control BG measurements from Figures 1A,B were analyzed for the change in blood glucose at the next measurement (whether the mice received PBS or were untreated). All the sCD137 treatments of seven sCD137 treated mice that did not progress to end stage T1D were assessed for effect on next treatment. There were *n* = 107 events in the control group and *n* = 64 in the sCD137 treated group. ^**^*p* < 0.0001, Mann-Whitney *U*-test.

Supplemental Figure 2 Soluble CD137 specifically binds to CD137 ligand; binding is blocked by anti-CD137L antibody. **(A)** Mouse CD137L expression on HEK293 cell line overexpressing mouse CD137 ligand (HEK-mCD137L) vs. control HEK293 cells. 2 ×10^6^ HEK-mCD137L or HEK293 cells were stained with anti-CD137L or isotype control Ab, followed with anti-Rat IgG2a biotin and streptavidin PE. Mouse CD137L expression was quantified by flow cytometry. One representative histogram of four independent experiments is shown. **(B,C)** sCD137 binding assay using HEK-mCD137L and recombinant sCD137. 2 ×10^6^ HEK-mCD137L or control HEK293 cells were preincubated with 1 μg/sample of anti-CD137L Ab or PBS, then incubated with titrated doses of sCD137 for 30 min at 4°C. Cell bound mouse sCD137 **(B,C)** was analyzed by flow (see methods). One representative histogram of two independent experiments is shown.

Supplemental Figure 3 Rapamycin and sCD137 comparably reduce cytokine expression in T cells. Splenic CD4+ T cells were magnetically isolated as above and stimulated with CD3/CD28 beads either alone, with sCD137, or with rapamycin. After 24 h, IL-2 (left panel) and IFN-ɤ (right panel) concentrations in the supernatant were measured by ELISA. Data expressed as mean/SEM from three biologically independent experimental samples. ^*^*p* < 0.05, ^**^*p* < 0.01, unpaired *t*-test.
