# Supplementary figures and images for: Soluble CD137 Ameliorates Acute Type 1 Diabetes by Inducing T Cell Anergy
Source: Front Immunol. 2019 Nov 7;10:2566. doi: 10.3389/fimmu.2019.02566 (PMC6853870; doi:10.3389/fimmu.2019.02566)

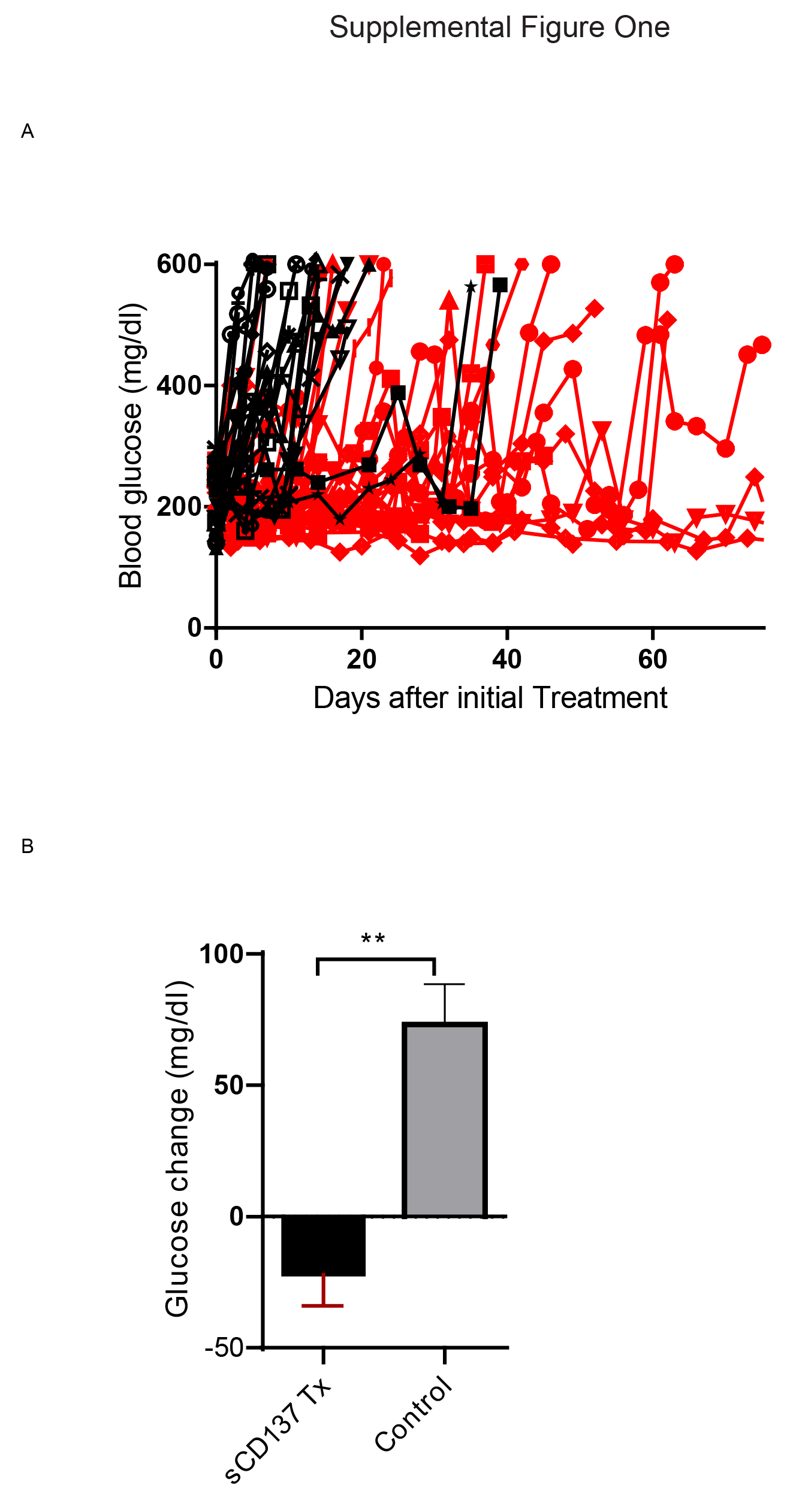

Supplement: Supplemental Figure 1 — Soluble CD137 treats acute T1D and induces treatment-related drops in serum blood glucose. (A) All individual blood glucose measurements from all the treated and control mice in Figures 1A,B. Control mice from Figures 1A,B are shown by black symbols/lines while sCD137 treated mice from Figures 1A,B are shown by red symbols/lines. (B) sCD137 reduces next blood glucose measurement compared to PBS/no treatment. All control BG measurements from Figures 1A,B were analyzed for the change in blood glucose at the next measurement (whether the mice received PBS or were untreated). All the sCD137 treatments of seven sCD137 treated mice that did not progress to end stage T1D were assessed for effect on next treatment. There were n = 107 events in the control group and n = 64 in the sCD137 treated group. **p < 0.0001, Mann-Whitney U-test. [file Image_1.TIF]

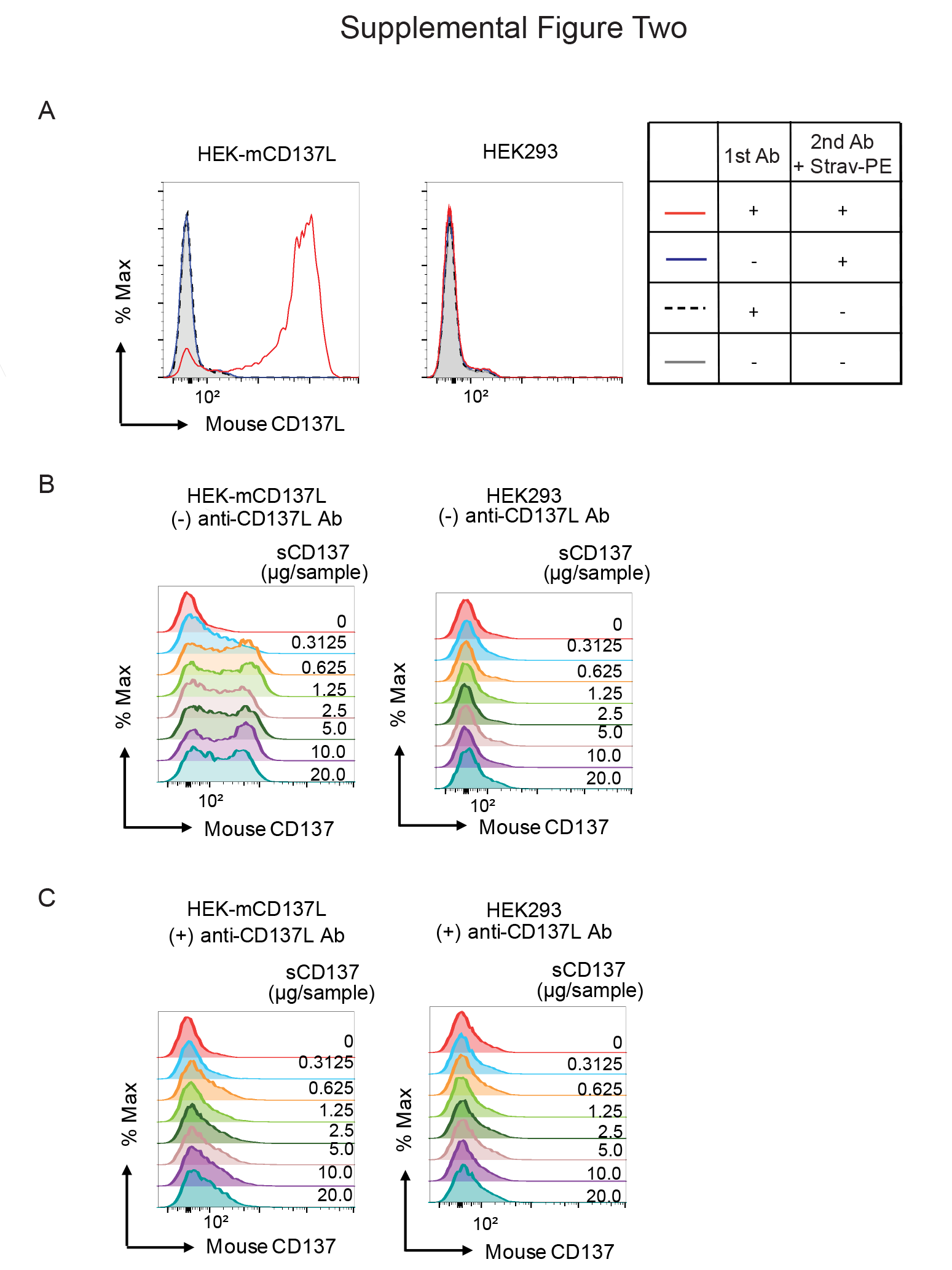

Supplement: Supplemental Figure 2 — Soluble CD137 specifically binds to CD137 ligand; binding is blocked by anti-CD137L antibody. (A) Mouse CD137L expression on HEK293 cell line overexpressing mouse CD137 ligand (HEK-mCD137L) vs. control HEK293 cells. 2 ×10^6^ HEK-mCD137L or HEK293 cells were stained with anti-CD137L or isotype control Ab, followed with anti-Rat IgG2a biotin and streptavidin PE. Mouse CD137L expression was quantified by flow cytometry. One representative histogram of four independent experiments is shown. (B,C) sCD137 binding assay using HEK-mCD137L and recombinant sCD137. 2 ×10^6^ HEK-mCD137L or control HEK293 cells were preincubated with 1 μg/sample of anti-CD137L Ab or PBS, then incubated with titrated doses of sCD137 for 30 min at 4°C. Cell bound mouse sCD137 (B,C) was analyzed by flow (see methods). One representative histogram of two independent experiments is shown. [file Image_2.TIF]

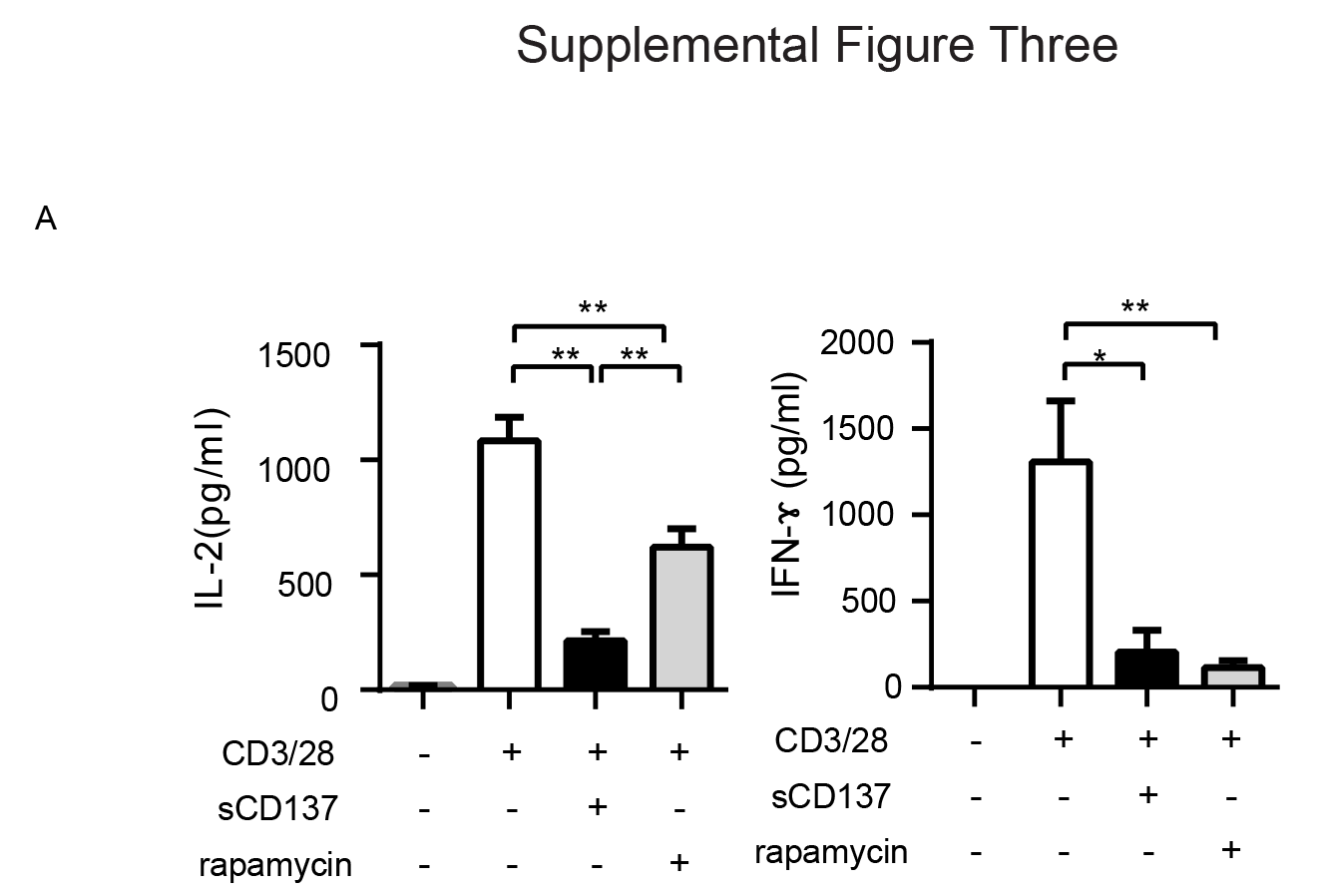

Supplement: Supplemental Figure 3 — Rapamycin and sCD137 comparably reduce cytokine expression in T cells. Splenic CD4+ T cells were magnetically isolated as above and stimulated with CD3/CD28 beads either alone, with sCD137, or with rapamycin. After 24 h, IL-2 (left panel) and IFN-ɤ (right panel) concentrations in the supernatant were measured by ELISA. Data expressed as mean/SEM from three biologically independent experimental samples. *p < 0.05, **p < 0.01, unpaired t-test. [file Image_3.TIF]
